# Saponins and Flavonoids from Adzuki Bean (*Vigna angularis* L.) Ameliorate High-Fat Diet-Induced Obesity in ICR Mice

**DOI:** 10.3389/fphar.2017.00687

**Published:** 2017-09-27

**Authors:** Rui Liu, Yinan Zheng, Zongwei Cai, Baojun Xu

**Affiliations:** ^1^Food Science and Technology Program, Beijing Normal University-Hong Kong Baptist University United International College, Zhuhai, China; ^2^Department of Chemistry, Hong Kong Baptist University, Kowloon Tong, China; ^3^College of Chinese Medicinal Materials, Jilin Agricultural University, Changchun, China

**Keywords:** adzuki bean, pancreatic lipase, lipolysis, obese mice, adipose tissue

## Abstract

**Background and purpose:** As an herbal medicine, adzuki bean has been practiced since the Tang Dynasty of China to maintain health and control weight; this practice is still very popular in China nowadays. However, it is still lack of sufficient scientific basis to explain scientific principle of this popular civil practice in weight control using adzuki bean. The purpose of this study was to verify and explain the anti-obesity effects of adzuki bean through *in vitro* enzymatic assays, *in vitro* lipolysis and *in vivo* study of obese mice model.

**Methods:** Inhibitory effects of flavonoids and saponins from adzuki bean (*Vigna angularis*) on pancreatic lipase, α-glucosidase activities, and noradrenaline-induced lipolysis were assessed. High-fat diet-induced obesity model was created to study anti-obesity effects of adzuki bean. Both serum and liver lipid parameters were determined after 8 weeks intervention.

**Results:** Adzuki bean extracts enhanced lipolysis. Compared to the final body weight of high-fat diet group, oral administration of adzuki bean significantly (*p* < 0.05) reduced the final body weight of mice and adipose tissue accumulation. The adzuki bean intervention also significantly reduced the levels of serum triglyceride, total cholesterol, low density lipoprotein-cholesterol, and liver lipid.

**Conclusion:** Adzuki bean demonstrated the anti-obesity effects on mice, such effects may mediated through the inhibitory effects of flavonoids and saponins from adzuki bean on α-glucosidase and pancreatic lipase activities, and lipolysis enhancement effect of active components from adzuki bean.

## Introduction

Overweight and obesity are defined by World Health Organization (WHO) as abnormal or excessive fat accumulation that presents a risk to health. It was reported that one billion adults were overweight, while more than 300 million were obese in the world in 2010 (WHO, 2010). Overweight and obesity lead to many serious diseases, such as high blood pressure, diabetes, strokes, cancers (Kushner, 2002; Festi et al., 2004; Nammi et al., 2004; WHO, 2010). Overweight and obesity are becoming a major public health issue globally.

Unhealthy diet is one of the major risk factors for chronic diseases like obesity and diabetes. It is widely accepted that dietary fat intake was directly or indirectly related to the occurrence of obesity, diabetes, high cholesterol, and other diseases (Hill et al., 2000). Consumption of bioactive compounds from diet or intake of dietary supplementation is one of possible ways to control obesity and to prevent or reduce the risks of developing various obesity-related diseases.

Food legumes are widely consumed by most nations, especially Asian countries like China. Saponins and phenolics from food legumes have been reported to possess many biological activities such as anti-inflammatory (Zha et al., 2011), anti-cancer (Zhang and Popovich, 2010), and anti-hypertension (Takahashi et al., 2008). Adzuki bean is mainly produced and consumed in China and several other countries in East Asia. It has been used as traditional Chinese herbal medicine and food for over thousands of years. The bioactivities, such as anti-tumor (Itoh et al., 2002, 2005), anti-diabetes (Itoh et al., 2004, 2009), and antioxidant (Wu et al., 2004; Yao et al., 2011a; Sreerama et al., 2012; Xu and Chang, 2012) have been documented previously.

“Eating beans to lose weight” has been practiced since the Tang Dynasty of China; currently “eating beans to lose weight” is still very popular folk practice in China, Japan, and South Korea. However, reports on health benefits of adzuki bean in obesity prevention or weight control are limited. It is still lack of sufficient scientific basis to explain scientific principle of these popular civil practices in weight control using adzuki bean. Therefore, the purpose of this study was to explain the anti-obesity effects of adzuki bean through *in vitro* enzymatic assays, *in vitro* lipolysis, and *in vivo* study of obese mice.

## Materials and Methods

### Materials

Adzuki beans (*Vigna angularis* L.) were purchased from local market in Changchun, Jilin Province, and identified by Prof. Jinming Mu of Faculty of Agronomy in Jilin Agricultural University. Triolein, taurocholic acid sodium salt hydrate, *N*- [Tris (hydroxymethyl) methyl]- 2- aminopropanesulfonic acid (TES) buffer, phosphatidylcholine, porcine pancreatic lipase, α-glucosidase, and *p*-nitrophenyl-α-glucopyranoside were purchased from Sigma (St. Louis, MO, United States). Lipid assay kits for testing triglycerides (TG), total cholesterol (TC), high density lipoprotein-cholesterol (HDL-C), and low density lipoprotein-cholesterol (LDL-C), were purchased from Biosino Bio-technology and Science Inc. (Beijing, China). Other chemicals, such as sodium dihydrogen phosphate dehydrate, disodium hydrogen phosphate dodecahydrate, were of analytical grade. Macroporous adsorptive resins AB-8 was supplied by Nankai University, and polyamide resin was purchased from Sinopharm Chemical Reagent Co., Ltd. (Beijing, China).

### Experimental Animals

Female ICR mice, 3 weeks old, were obtained from the Experimental Animal Holding Center of Jilin University, and housed individually in plastic cages in a 12/12 h light/dark cycle in a temperature- and humidity-controlled room for 1 week adaptation. The animals were allowed to access food and water freely. The healthy ones were used for further anti-obesity study. Young male Wistar rats (5 weeks old) were also obtained from the Experimental Animal Holding Center of Jilin University, which were utilized to carry out the *in vitro* lipolysis experiments. The Animal Care and Use Committee of Jilin Agricultural University approved all experimental procedures. This study was conducted according to the National Research Council’s Guide for the Care and Use of Laboratory Animals.

### Diets for ICR Mice

Both normal diet (ND) and high-fat diet (HFD) were purchased from the feed center of the Experimental Animal Holding Center of Jilin University (Changchun, China). ND consisted of 5% fat, 53% carbohydrate, 23% protein, and total calorific value 25 kJ/kg. HFD consisted of 22% fat, 48% carbohydrate, and 20% protein, and total calorific value 44.3 kJ/kg. The diets were stored at -20°C and prepared freshly each day to avoid auto-oxidation of lipids.

### Preparation of Flavonoids and Saponins from Adzuki Bean

Flavonoids of adzuki bean (ABF) and saponins of adzuki bean (ABS) were prepared according to the previous articles (Jia and Lu, 2008; Itoh and Furuichi, 2009). Briefly, adzuki bean was ground and 14 kg of the powder was then extracted with 140 L of 70% ethanol for three times. The combined extract solution was filtrated and concentrated to remove ethanol. The remaining aqueous solution was extracted with 14 L of petroleum ether at room temperature for three times. The aqueous phase was then extracted with 14 L of *n*-butanol at room temperature three times. The *n*-butanol solution was evaporated under vacuum to obtain 158.6 g of *n*-butanol extract which was total extract of adzuki bean (ABTE). The crude flavonoids were collected in the 45% ethanol fraction from AB-8 resin column after eluting with water. The collected ABF were subjected to the second column with polyamide according to the literatures (Wu et al., 2009; Wei et al., 2011), and the enriched flavonoid was further obtained in 40% ethanol fraction after eluting with 10% ethanol from polyamide column. ABF was obtained from the supernatant after precipitating with methanol–acetone finally. The enriched ABS was collected in the 80% ethanol fraction after eluting with 45% ethanol by AB-8 resin column. With further precipitation method, ABS was precipitated by the reagents of methanol–acetone. The chemical constituents of ABTE, ABF, and ABS were identified by liquid chromatography-ion trap mass spectrometry. Quantitative analysis was performed on an Agilent 1100 series HPLC system. The contents of saponins and flavonoids were reported in our latest publication (Liu et al., 2017).

### α-Glucosidase Activity Assay

α-Glucosidase inhibitory activity of adzuki bean was investigated according to the method of Kwon et al. (2008). Briefly, a volume of 50 μL of different concentrations of sample solutions and 100 μL of 0.1 M phosphate buffer (pH 6.9) containing α-glucosidase solution (1.0 U/mL) were incubated in a 96-well plate at 25°C for 10 min. After pre-incubation, 50 μL of 5 mM *p*-nitrophenyl-α-D-glucopyranoside solution in 0.1 M phosphate buffer (pH 6.9) was added to each well at 5 s intervals. The reaction mixtures were incubated at 25°C for 5 min. Absorbance readings were recorded at 405 nm by a microplate reader (FLUOstar Omega 415-1179, BMG LABTECH GmbH, Germany), and the absorbance of a control which contained 50 μL of buffer solution instead of the extract was recorded under the same experimental conditions. The % inhibition of α-glucosidase activity was calculated as the following equation:

$$\%\;\mathrm{Inhibition}=\left(\frac{A_{405}^{\mathrm{Control}}-A_{405}^{\mathrm{Sample}}}{A_{405}^{\mathrm{Control}}}\times 100\right).$$

### Pancreatic Lipase Activity Assay

Lipase activity was determined according to the previous articles (Zapf et al., 1981; Tsujita and Okuda, 1983) with a slight modification. A suspension of triolein 80 mg, phosphatidylcholine 10 mg, and taurocholic acid 5 mg in 9 mL of 0.1 M TES buffer (pH 7.0) containing 0.1 M NaCl was sonicated for 10 min by a KQ 5200 DE ultrasonic equipment (Kunshan Ultrasonic Instruments Co., Ltd, Jiangsu, China). This sonicated substrate suspension 10 μL was incubated with 50 μL of pancreatic lipase and 100 μL of various concentrations of chondroitin sulfate solution for 30 min at 37°C in a final volume of 250 μL. The incubation mixture was added into 3 mL aliquots of a 1:1 (v/v) mixture of chloroform and *n*-heptane containing 2% (v/v) methanol and extracted by shaking the tubes horizontally for 10 min by a vortex (XK96-A, Xinkang Medical Apparatus Co., Ltd., Jiangsu, China). The mixture wascentrifuged at3000 rpm for 10 min (TG16-WS centrifuge, Hunan, China), and the upper aqueous phase was removed by suction. Copper reagent (1 mL) was then added to the lower organic phase. The tube was shaken for 10 min, the mixture was centrifuged at 3000 rpm for 10 min, and then 1.5 mL of the upper organic phase, which contained copper salts of the extracted free fatty acids, was treated with 1.5 mL of 0.1% (w/v) bathocuproine in chloroform containing 0.05% (w/v) 3(2)-*tert*-butyl-4-hydroxyanisole. The absorbance was then measured at 480 nm by 722-visible spectrophotometer (Shanghai, China). The % inhibition of pancreatic lipase activity was calculated as the following equation:

$$\%\;\mathrm{Inhibition}=\left(\frac{A_{480}^{\mathrm{Control}}-A_{480}^{\mathrm{Sample}}}{A_{480}^{\mathrm{Control}}}\times 100\right).$$

### Lipolysis Assay

Young male Wistar rats (150–160 g) were given a standard laboratory ND and water freely. The rats were kept in a 12/12 h light/dark cycle in a temperature- and humidity-controlled room. Then the rats were killed by cervical dislocation and their epididymal adipose tissues were quickly collected. Fat cells were isolated from the epididymal adipose tissue according to a previous article (Rodbell, 1964). Briefly, the epididymal fat pads excluding the vascular were rinsed in Hank’s buffer, and dried by filter paper. Thin distal portions from each epididymal fat pad were cut into several small pieces with scissors. Collagenase solution was added to the minced tissue pieces, and incubated at 37°C for 1 h. After incubation, the tissue was dispersed into small fragments, and filtered. The filtrate was centrifuged at 1000 rpm, and finally the fat cells were collected from the upper layer.

The fat cell fraction was incubated for 1 h at 37°C in Hank’s balanced solution (pH 7.4) supplemented with 2.5% bovine serum albumin, noradrenaline, and the indicated amounts of tests. According to the previous article (Okuda et al., 1986), the release of free fatty acid was measured. Briefly, the incubation mixture (250 μL) was mixed with 3 mL of chloroform-*n*-heptane (1:1, v/v) containing 2% methanol and extracted by shaking the tube horizontally for 10 min in a shaker. The mixture was centrifuged at 3000 rpm at 25°C for 5 min, and the upper aqueous phase was removed by suction. Copper reagent (1 mL) was then added to the lower organic phase. Then the tube was shaken for 10 min, the mixture was centrifuged at 3000 rpm at 25°C for 10 min, and 1.5 mL of the upper organic phase was treated with 1.5 ml of 0.1% (w/v) bathocuproine in chloroform containing 0.05% (w/v) 3(2)-*tert*-butyl-*4*-hydroxyanisole. The absorbance was then measured at 480 nm by 722-visible spectrophotometer (Shanghai, China). The effects of lipolytic relative to control and standard noradrenaline were calculated as the following equation:

$$\%\;\mathrm{Lipolytic}=\frac{\left(A_{480}^{\mathrm{Sample}}-A_{480}^{\mathrm{Control}}\right)-\left(A_{480}^{\mathrm{Standard}}-A_{480}^{\mathrm{Control}}\right)}{A_{480}^{\mathrm{Standard}}-A_{480}^{\mathrm{Control}}}\times 100.$$

### Estimation of Relative Parameters of Mice Fed with a High-Fat Diet

After adaptation to the ND and the new life condition for 1 week, the healthy mice were randomly divided into eight groups (one normal group, seven HFD groups) according to the weight, of eight mice each group. The normal group was fed with ND and other seven groups were fed with a HFD for 4 weeks. Then the ND group was constantly fed with ND and the mice were orally administered physiological saline per day for additional 4 weeks. The HFD groups were treated with different ways for additional 4 weeks and were further divided into HFD group treated with physiological saline, HFD group orally administered with ABTE of 60 mg/kg per day (HFD + ABTE60), HFD group with ABTE of 300 mg/kg (HFD + ABTE300), HFD group with ABF of 60 mg/kg (HFD + ABF60), HFD group with ABF of 300 mg/kg (HFD + ABF300), HFD group with ABS of 60 mg/kg (HFD + ABS60), and HFD group with ABS of 300 mg/kg (HFD + ABS300).

After 8 weeks of different treatments, blood was taken from each mouse by the artery and vein of fossa orbitalis (Liu et al., 2010). Then the blood was centrifuged at 4°C and the serum of each mouse was obtained and frozen at -80°C until further analysis. Serum TG, TC, HDL-C, and LDL-C were determined using the lipid assay kits by BS-400 Clinical Chemistry Analyzer (Mindray, Shenzhen, China), respectively.

After sampling blood, mice were killed by cervical fracture. The heart, liver, spleen, kidney, thymus, and adipose tissues (parametrial plus peritoneal and perirenal adipose tissue) were quickly sampled and weighed. The liver tissues were stored at -80°C until further analysis. The liver TG and TC concentrations were measured by the following method: 0.5 g of the liver tissue was homogenized in 4.5 mL of Krebs Ringer phosphate buffer (pH 7.4), then 0.2 mL of the homogenate was extracted with 4 mL chloroform/methanol (2:1, v/v), the extract was concentrated under a nitrogen stream. The dry residue was analyzed using TG and TC test kits.

### Statistical Analysis

The values were expressed as means ± standard errors (SE). Statistical analysis was calculated by ANOVA using SPSS software (version 21). Scheffe’s test was used to analyze the data. The criterion for statistical significance was *p* < 0.05.

## Results and Discussion

### Characterization and Quantification of Saponins and Flavonoids in Adzuki Bean

The HPLC results revealed the presence of flavonoids mainly in 45% ethanol fractions and the presence of saponins mainly in 80% ethanol fractions by AB-8 resin column. It was reported that AB-8 resin was good at separating chemical constituent according to the polarity (Liu et al., 2010). After that, polyamide column was employed to further purify the flavonoids. Eluent of 40% ethanol from polyamide column was obtained, and the fraction was rich in flavonoids. Precipitation with methanol–acetone was applied to further separate flavonoids and saponins from adzuki bean. Flavonoids existed in the supernatant fraction, while saponins presented in the precipitate fraction. Finally, ABTE, ABF, and ABS were obtained and utilized for HPLC-DAD-ESI-MS^n^ analysis. The detailed characterization of compounds in adzuki bean extract was presented in author thesis (Liu, 2014). The contents of saponins and flavonoids in adzuki bean extract were reported in our latest publication (Liu et al., 2017). Briefly, the contents of catechin (49.4 mg/g), quercetin-3-*O*-rutinoside (404.7 mg/g), quercetin-3-*O*-glucoside (90.1 mg/g), and vitexin-4″-*O*-glucoside (74.6 mg/g) in ABF extract were much higher than that of the ABTE (12.4, 225.9, 21.4, and 36.7 mg/g, respectively). Meanwhile, the contents of azukisaponin IV (11.4 mg/g), azukisaponin VI (206.3 mg/g), azukisaponin V (283.2 mg/g), azukisaponin II (389.7 mg/g), azukisaponin I (5.4 mg/g), and azukisaponin III (27.6 mg/g) in adzuki bean saponins extract were much higher than that of ABTE (6.6, 20.0, 165.9, 186.9, 8.9, and 79.0 mg/g, respectively).

### Effects of Adzuki Bean on α-Glucosidase and Pancreatic Lipase Activities *In Vitro*

α-Glucosidase is a key enzyme to hydrolysis polysaccharides and disaccharides into glucose in small intestine (Vannasaeng et al., 1995; Mooradian and Thurman, 1999; Scheen, 2003). Inhibition of α-glucosidase activity can block digestion and absorption of carbohydrates, further control metabolism disorders such as diabetes and obesity; while pancreatic lipase is a key enzyme to catalyze the hydrolysis of dietary fat in the digestive system (Mukherjee, 2003), 50–70% of the dietary fat can be hydrolyzed by this enzyme. Thus inhibition of pancreatic lipase activity can block fat absorption in gastrointestinal tract, further control obesity incidence, especially diet-induced obesity. The previous articles also reported that adzuki bean had the inhibitory effects on the two enzymes (Yao et al., 2011a,b; Sreerama et al., 2012). Yao et al. (2011b) tested α-glucosidase activity of 16 food legumes, and the results showed that adzuki bean had the highest inhibition activity. Further study of different fractions of 70% ethanol extract of adzuki bean also exhibited inhibitory effects on α-glucosidase. In the present study, total extract, flavonoids, and saponins from adzuki bean demonstrated inhibitory effects on both α-glucosidase and pancreatic lipase.

Total extract, flavonoids, and saponins from adzuki bean demonstrated inhibitory effects on both α-glucosidase and pancreatic lipase in dose-dependent manner at the concentration ranged from 0.25 to 1 mg/mL in the assay system using triolein emulsified with phosphatidylcholine (**Table 1**). ABF (1 mg/mL) exhibited the highest inhibitory rate of α-glucosidase with 91.5%, followed by saponin (68.3%), and total extract (55.7%). The inhibitory rates of α-glucosidase were respectively much higher than 50% except for the ABTE at the concentration of 0.25 mg/mL. With regard to pancreatic lipase activity, ABTE, ABF, and ABS exhibited the similar inhibitory rates (around 40%).

**Table 1:** Effect of adzuki bean on α-glucosidase and pancreatic lipase activities.

Concentration of	α-Glucosidase	Pancreatic lipase
samples (mg/mL)	inhibition (%)	inhibition (%)
**Control**		
0	0 ± 0^a^	0 ± 0^a^
**ABTE**		
0.25	48.69 ± 0.513^b^	23.77 ± 2.037^b^
0.50	50.67 ± 0.443^c^	29.25 ± 2.673^bc^
0.75	52.74 ± 0.208^d^	36.40 ± 1.777^de^
1.00	55.65 ± 0.227^e^	42.90 ± 0.809^e^
**ABF**		
0.25	81.74 ± 0.201^f^	38.63 ± 2.215^bc^
0.50	87.09 ± 0.387^g^	29.34 ± 1.625^c^
0.75	88.52 ± 0.277^g^	33.89 ± 2.727^de^
1.00	91.84 ± 0.288^h^	39.65 ± 1.161^e^
**ABS**		
0.25	57.63 ± 0.218^i^	21.17 ± 1.114^b^
0.50	62.84 ± 0.254^j^	25.07 ± 1.163^bc^
0.75	67.50 ± 0.599^k^	39.83 ± 1.214^e^
1.00	68.33 ± 0.092^k^	42.80 ± 1.810^e^

*ABTE, total extract of adzuki bean; ABF, flavonoids of adzuki bean; ABS, saponins of adzuki bean. Values are expressed as means ± SE of three experiments. Values marked by the same letters within column are not significantly different (*p* < 0.05).*

### Effects of Adzuki Bean on Noradrenaline-Induced Lipolysis of Isolated Fat Cells from Rats

Lipolysis assay was carried out in the present study according to the previous articles (Okuda et al., 1986; Han et al., 1999). The present article showed that ABTE, ABF, and ABS enhanced the noradrenaline-induced lipolysis (166.1, 175.6, and 152.6%, respectively) in the isolated fat cells at the concentration of 1 mg/mL (**Table 2**). While ABTE, ABF, and ABS without noradrenaline also enhanced lipolysis (136.8, 112.9, and 119.7%, respectively) in the fat cells at the same concentration. In a word, ABTE, ABF, and ABS enhanced the noradrenaline-induced lipolysis in the isolated fat cells. It implied that adzuki bean could contribute to weight lose by breaking down fats.

**Table 2:** Effect of adzuki bean on noradrenaline-induced lipolysis in isolated fat cells from rats.

	Lipolysis (% of control)
Treatments	Mean ± SE (*n* = 3)
None	0 ± 0^a^
Noradrenaline (0.01 μg/mL)	100 ± 0^b^
Noradrenaline + ABTE (1 mg/mL)	166.08 ± 4.094^ef^
ABTE (1 mg/mL)	136.84 ± 7.542^d^
Noradrenaline + ABF (1 mg/mL)	175.63 ± 7.935^f^
ABF (1 mg/mL)	112.87 ± 3.094^bc^
Noradrenaline + ABS (1 mg/mL)	152.63 ± 5.916^e^
ABS (1 mg/mL)	119.69 ± 3.514^c^

*ABTE, total extract of adzuki bean; ABF, flavonoids of adzuki bean; ABS, saponins of adzuki bean. Values are expressed as means ± SE of three experiments. Values marked by the same letters within column are not significantly different (*p* < 0.05).*

### Effects of Adzuki Bean on Body Weight of Mice Fed with a High-Fat Diet for 8 Weeks

**FIGURE 1:**
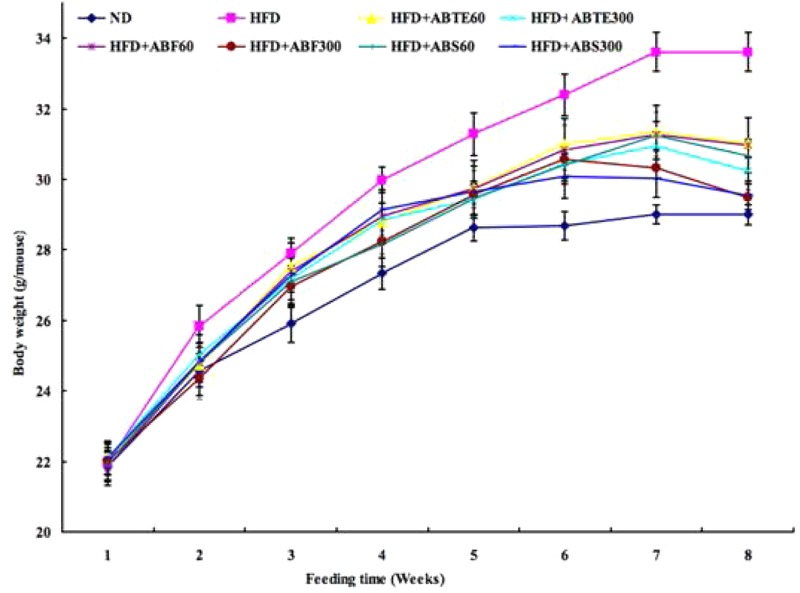
Effect of adzuki bean on body weight of mice fed with a high-fat diet for 8 weeks. ND, normal diet group; HFD, high-fat diet group; HFD + ABTE60, HFD group orally administered total extract of adzuki bean of 60 mg/kg per day; HFD + ABTE300, HFD group orally administered total extract of adzuki bean of 300 mg/kg per day; HFD + ABF60, HFD group with ABF of 60 mg/kg; HFD + ABF300, HFD group with ABF of 300 mg/kg; HFD + ABS60, HFD group with ABS of 60 mg/kg; HFD + ABS300, HFD group with ABS of 300 mg/kg. Values are expressed as means ± SE (*n* = 8). Values marked by the same letters within final body weight are not significantly different (*p* < 0.05).

In order to investigate the effects of adzuki bean on obesity in mice fed with HFD, body weight was recorded during the 8 weeks and set to the first parameter to evaluate obesity. The kinetic changes of body weight of mice with different treatments during 8 weeks were presented in **Figure 1**. There was a whole increase trend of body weights; however, there were significant (*p* < 0.05) differences between the HFD group and other groups with or without treatment of ABTE, ABF, or ABS. After 8 weeks, HFD increased the body weights of HFD group mice from 21.96 to 33.61 g, while body weights of ND group mice increased from 21.86 to 29.00 g. Compared to the HFD group, ABTE treatments (300 mg/kg per day) significantly (*p* < 0.05) reduced the final body weight by 7.7%, while ABTE treatments (300 mg/kg per day) reduced the final body weight by 10.0%. ABF and ABS treatments also revealed significant reduction of the final body weight as compared to the HFD group, especially ABF (300 mg/kg per day) treatment with reduction of the final body weight by 12.2%. ABTE, ABF, or ABS reduced the mice weights. Wu et al. (2011) reported that alcohol extract of adzuki bean reduced the body weight of male and female Kunming mice fed with a HFD, which supported our present results that adzuki bean could reduce body weight of ICR female mice fed with a HFD. Additionally, adzuki bean seed coats could significantly reduce body weight of streptozotocin-induced diabetic rats (Sato et al., 2005), while hot water extract of adzuki bean also could reduce body weights of rats (Itoh and Furuichi, 2009).

### Effects of Adzuki Bean on Organ Weight and Adipose Tissue Weight of Obese Mice

Thymus weight, liver weight, spleen weight, and kidney weight of mice with different treatments were analyzed deliberatively after 8 weeks (**Table 3**). Adzuki bean treatments increased the thymus weight and decreased the liver weight as compared to the HFD group. The weight of spleen with or without adzuki bean treatment showed no differences in the present study. With regard to kidney, ABTE, ABF, and ABS could reduce kidney weight as compared to HFD group mice, and the ABF and ABS exhibited the better effect, the kidney weight in the mice treated with flavonoids and saponins were almost similar to that of ND group mice.

**Table 3:** Effect of adzuki bean on organ weights of mice fed with a high-fat diet for 8 weeks.

	Final body	Thymus	Liver	Spleen	Kidney
Groups	weight (g/mouse)	(g/mouse)	(g/mouse)	(g/mouse)	(g/mouse)
ND	29.00 ± 0.284^a^	0.085 ± 0.004^b^	1.156 ± 0.056^a^	0.126 ± 0.003^a^	0.296 ± 0.009^a^
HFD	33.61 ± 0.544^c^	0.065 ± 0.004^a^	1.947 ± 0.097^d^	0.119 ± 0.004^a^	0.336 ± 0.013^b^
HFD + ABTE60	31.03 ± 0.722^a^	0.067 ± 0.004^a^	1.485 ± 0.036^c^	0.121 ± 0.008^a^	0.302 ± 0.014^ab^
HFD + ABTE300	30.24 ± 0.366^ab^	0.075 ± 0.005^ab^	1.401 ± 0.031^bc^	0.126 ± 0.003^a^	0.304 ± 0.013^ab^
HFD + ABF60	30.97 ± 0.779^b^	0.067 ± 0.006^a^	1.238 ± 0.018^a^	0.127 ± 0.003^a^	0.292 ± 0.009^a^
HFD + ABF300	29.50 ± 0.193^ab^	0.072 ± 0.007^ab^	1.176 ± 0.048^a^	0.123 ± 0.005^a^	0.282 ± 0.015^a^
HFD + ABS60	30.67 ± 0.472^b^	0.068 ± 0.009^ab^	1.317 ± 0.054^ab^	0.123 ± 0.007^a^	0.294 ± 0.009^a^
HFD + ABS300	29.55 ± 0.406^ab^	0.070 ± 0.004^ab^	1.162 ± 0.030^a^	0.128 ± 0.007^a^	0.279 ± 0.010^a^

*ND, normal diet group; HFD, high-fat diet group; HFD + ABTE60, HFD group orally administered total extract of adzuki bean of 60 mg/kg per day; HFD + ABTE300, HFD group orally administered total extract of adzuki bean of 300 mg/kg per day; HFD + ABF60, HFD group with ABF of 60 mg/kg; HFD + ABF300, HFD group with ABF of 300 mg/kg; HFD + ABS60, HFD group with ABS of 60 mg/kg; HFD + ABS300, HFD group with ABS of 300 mg/kg. Values are expressed as means ± SE (*n* = 8). Values marked by the same letters within column are not significantly different (*p* < 0.05).*

**FIGURE 2:**
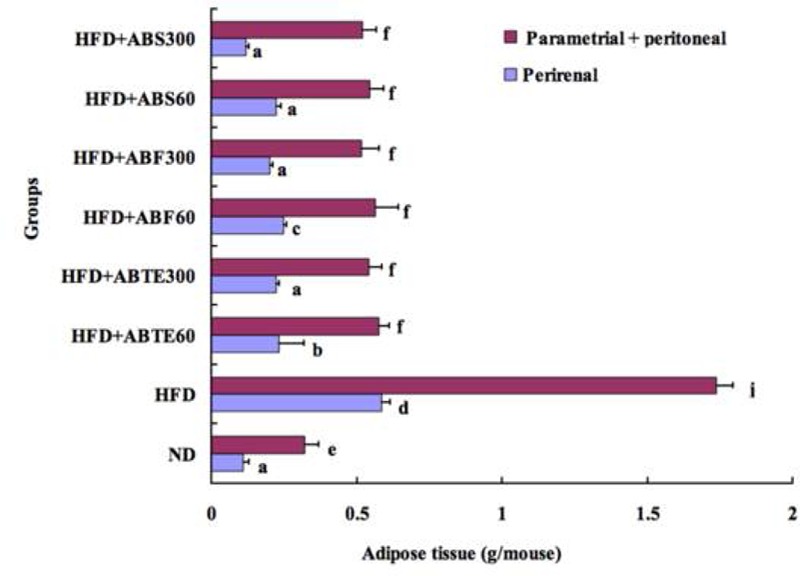
Effect of adzuki bean on parametrial and perirenal adipose tissue weight of mice fed with a high-fat diet for 8 weeks. ND, normal diet group; HFD, high-fat diet group; HFD + ABTE60, HFD group orally administered total extract of adzuki bean of 60 mg/kg per day; HFD + ABTE300, HFD group orally administered total extract of adzuki bean of 300 mg/kg per day; HFD + ABF60, HFD group with ABF of 60 mg/kg; HFD + ABF300, HFD group with ABF of 300 mg/kg; HFD + ABS60, HFD group with ABS of 60 mg/kg; HFD + ABS300, HFD group with ABS of 300 mg/kg. Values are expressed as means ± SE (*n* = 8). Values marked by the same letters within parametrial or perirenal adipose tissue weight are not significantly different (*p* < 0.05).

Body fat, especially abdominal fat, is harmful to human health. A previous article had reported that adzuki bean decreased adipose tissue weights in mice and rats (Wu et al., 2011). Our results verified the previous findings. The bar chat (**Figure 2**) compared perirenal and parametrial plus peritoneal adipose tissue weights among the mice groups with different treatments. Feeding a HFD for 8 weeks caused significant (*p* < 0.05) increases tissue weight in both perirenal and parametrial plus peritoneal adipose tissue as compared to the ND group. The perirenal and parametrial plus peritoneal adipose tissue weights of HFD group were 0.59 and 1.74 g, respectively, while that of ND group were 0.11 and 0.32 g. Adzuki bean treatment reduced significantly both perirenal and parametrial plus peritoneal adipose tissue weights as compared to the HFD group. It was an increase trend with the increased dose of ABTE, ABF, or ABS. Saponins treatment (300 mg/kg per day) demonstrated the best effect in terms of reducing perirenal and parametrial plus peritoneal adipose tissue weights. The perirenal and parametrial plus peritoneal adipose tissue weights of mice treated with ABS (300 mg/kg per day) were 0.12 and 0.52 g, respectively.

### Effects of Adzuki Bean on Biochemical Parameters of Serum and Liver of Obese Mice

As the main risk factors for dyslipidemia (Klein, 2004), serum TC and TG was considered as unhealthy symbols of human body. In the present study, the HFD significantly (*p* < 0.05) induced fasting serum concentrations of serum TG, TC, and LDL-C, as compared to mice treated with the ND (**Table 4**). Adzuki bean treatment with the HFD suppressed the increases of serum TG, TC, and LDL-C concentrations as compared to the HFD group without adzuki bean treatment, while increased the level of HDL-C. The higher dose of adzuki bean samples, the more decreases in serum TG, TC, and LDL-C concentrations, while more increases in serum HDL-C. Wu et al. (2011) also pointed out that adzuki bean lowered blood TG and TC levels and increased HDL-C level of Kunming mice. Adzuki bean also decreased serum TC and TG levels of rats; however, it did not increase HDL-C level of rats (Itoh and Furuichi, 2009; Kitano-Okada et al., 2012).

**Table 4:** Effect of adzuki bean on serum parameters and liver parameters after 8 weeks.

	Serum parameters				Liver parameters	
Groups	TG (mmol/L)	TC (mmol/L)	HDL-C (mmol/L)	LDL-C (mmol/L)	TG (μmol/g liver)	TC (μmol/g liver)
ND	1.27 ± 0.031^ab^	2.86 ± 0.089^a^	3.17 ± 0.122^c^	0.35 ± 0.029^a^	6.02 ± 0.049^a^	4.13 ± 0.053^a^
HFD	2.43 ± 0.067^e^	4.89 ± 0.129^f^	2.20 ± 0.088^a^	0.77 ± 0.034^e^	18.00 ± 0.544^d^	8.26 ± 0.073^e^
HFD + ABTE60	1.53 ± 0.067^d^	4.19 ± 0.089^e^	2.64 ± 0.064^b^	0.68 ± 0.037^d^	14.89 ± 0.351^c^	6.51 ± 0.111^d^
HFD + ABTE300	1.31 ± 0.046^ab^	4.02 ± 0.086^cde^	2.66 ± 0.102^b^	0.60 ± 0.025^c^	11.97 ± 0.119^b^	5.43 ± 0.125^b^
HFD + ABF60	1.29 ± 0.025^ab^	4.16 ± 0.052^de^	2.78 ± 0.039^b^	0.69 ± 0.024^de^	14.82 ± 0.368^c^	6.68 ± 0.060^d^
HFD + ABF300	1.17 ± 0.040^a^	3.75 ± 0.028^b^	2.83 ± 0.038^b^	0.53 ± 0.018^bc^	11.56 ± 0.109^b^	5.38 ± 0.101^b^
HFD + ABS60	1.47 ± 0.046^cd^	3.91 ± 0.074^bcd^	2.67 ± 0.082^b^	0.70 ± 0.024^de^	14.34 ± 0.141^c^	6.65 ± 0.065^d^
HFD + ABS300	1.34 ± 0.020^bc^	3.89 ± 0.074^bc^	2.69 ± 0.067^b^	0.50 ± 0.016^b^	11.22 ± 0.068^b^	5.74 ± 0.099^c^

*Values are expressed as means ± SE (*n* = 8). Values marked by the same letters within column are not significantly different (*p* < 0.05). ND, normal diet group; HFD, high-fat diet group; HFD + ABTE60, HFD group orally administered total extract of adzuki bean of 60 mg/kg per day; HFD + ABTE300, HFD group orally administered total extract of adzuki bean of 300 mg/kg per day; HFD + ABF60, HFD group with ABF of 60 mg/kg; HFD + ABF300, HFD group with ABF of 300 mg/kg; HFD + ABS60, HFD group with ABS of 60 mg/kg; HFD + ABS300, HFD group with ABS of 300 mg/kg; TG, triglycerides; TC, total cholesterol; HDL-C, high density lipoprotein-cholesterol.*

Feeding a HFD caused mice liver to accumulate higher content of TG (18.00 μmol/g liver) and TC (8.26 μmol/g liver) as compared to the ND group (6.02 μmol TG/g liver and 4.13 μmol TC/g liver, respectively). The concentration of liver TG was about threefold higher than that of the normal group, while liver TC was over twofold higher than that of the normal group. Adzuki bean treatments, especially the high dose, retarded the accumulation of liver TG and TC, but did not recover to the normal level.

## Conclusion

In summary, *in vitro* and *in vivo* anti-obesity effects of total extract, flavonoids, and saponins extracted from adzuki bean were respectively investigated in the present study. The results indicated that total extract, flavonoids, and saponins possessed the inhibitory effects against pancreatic lipase and α-glucosidase, while flavonoids and saponins enhanced the noradrenaline-induced lipolysis in fat cells. The animal study also demonstrated the anti-obesity ability of these components. Our results showed that total extract, flavonoids, and saponins from adzuki bean could decrease body weight, adipose tissue weights, serum TG, TC, and LDL-C, and live lipids of mice fed with a HFD while increase serum HDL-C by orally administered method. The results of the present study indicate that adzuki bean is a potential important medicinal source for the prevention of obesity and the related diseases. The limitation of the current study was that the crude extracts were used, because there were no enough amounts of isolated and purified compounds from adzuki bean for animal study. Therefore, further studies should to be done in compounds isolation and be focus on the anti-obesity molecular mechanism of bioactive compounds from adzuki bean by cell models and the anti-obesity effects through clinical trials. In addition, new anti-obesity products derived from adzuki bean deserve to be developed in the future.

## Author Contributions

RL designed, carried out experiments and wrote the manuscript. YZ helped in animal feeding, dissection and carryout few biochemical assays. ZC designed, supervised, and corrected the manuscript. BX designed, wrote, and corrected the manuscript.

## Conflict of Interest Statement

The authors declare that the research was conducted in the absence of any commercial or financial relationships that could be construed as a potential conflict of interest.
